# Development and Validation of a Stability-Indicating UPLC Method for the Determination of Hexoprenaline in Injectable Dosage Form Using AQbD Principles

**DOI:** 10.3390/molecules26216597

**Published:** 2021-10-31

**Authors:** Jesús Alberto Afonso Urich, Viktoria Marko, Katharina Boehm, Raymar Andreína Lara García, Dalibor Jeremic, Amrit Paudel

**Affiliations:** 1 Research Center Pharmaceutical Engineering GmbH, 8010 Graz, Austria; jesus.afonso@rcpe.at (J.A.A.U.); viktoria.marko@rcpe.at (V.M.); katharina.boehm@rcpe.at (K.B.); lara.raymar@rcpe.at (R.A.L.G.); 2 Department of Health Studies-Biomedical Science, FH JOANNEUM, 8020 Graz, Austria; dalibor.jeremic@fh-joanneum.at; 3 Institute of Process and Particle Engineering, Graz University of Technology, 8010 Graz, Austria

**Keywords:** hexoprenaline, analytical quality by design (AQbD), design of experiment, stability-indicating method, ultra-performance liquid chromatography

## Abstract

A novel and efficient stability-indicating, reverse phase ultra-performance liquid chromatographic (UPLC^®^) analytical method was developed and validated for the determination of hexoprenaline in an injectable dosage form. The development of the method was performed using analytical quality by design (AQbD) principles, which are aligned with the future requirements from the regulatory agencies using AQbD principles. The method was developed by assessing the impact of ion pairing, the chromatographic column, pH and gradient elution. The development was achieved with a Waters Acquity HSS T3 (50 × 2.1 mm i.d., 1.8 µm) column at ambient temperature, using sodium dihydrogen phosphate 5 mM + octane-1-sulphonic acid sodium salt 10 mM buffer pH 3.0 (Solution A) and acetonitrile (Solution B) as mobile phases in gradient elution (t = 0 min, 5% B; t = 1 min, 5% B; t = 5 min, 50% B; t = 7 min, 5% B; t = 10 min, 5% B) at a flow rate of 0.5 mL/min and UV detection of 280 nm. The linearity was proven for hexoprenaline over a concentration range of 3.50–6.50 µg/mL (R^2^ = 0.9998). Forced degradation studies were performed by subjecting the samples to hydrolytic (acid and base), oxidative, and thermal stress conditions. Standard solution stability was also performed. The proposed validated method was successfully used for the quantitative analysis of bulk, stability and injectable dosage form samples of the desired drug product. Using the AQbD principles, it is possible to generate methodologies with enhanced knowledge, which can eventually lead to a reduced regulatory risk, high quality data and lower operational costs.

## 1. Introduction

Hexoprenaline sulphate is a selective β_2_-adrenoreceptor agonist that is used as a bronchodilator by oral or intravenous routes and by inhalation [1], and is indicated for the use in the treatment of bronchospasm associated with obstructive airways diseases, including asthma, bronchitis and emphysema [1,2]. Additionally, there have been recent findings of its clinical use as a tocolytic agent [3,4,5,6,7,8]. Hexoprenaline has been proven to have a comparable response and tolerability to other tocolytic drugs, with no major negative effects on the short and long term neonatal outcome [9,10].

Hexoprenaline is marketed as a free base, but also as a dihydrochloride or sulphate salt. The oral and injectable dosage form of hexoprenaline, even if is not widely approved in all major markets, has some approved marketing authorizations among some members states of the European Union [11]. At the moment, there is no compendial analytical methodology for the characterization of the molecule, and the number of analytical methodologies published is also limited. There is one assay method of hexoprenaline reported using potentiometry [12]. However, this analytical technique lacks the method specificity needed to assess the stability samples of the product. When a nonspecific assay methodology is used for the control of pharmaceuticals, it must be justified in combination with other supporting analytical procedures in order to ensure that any interfering species can be detected [13,14]. Thus, there is a lack of analytical methodologies published with a stability-indicating feature for hexoprenaline together with the current and upcoming regulatory requirements [15,16,17].

Quality by design (QbD) is defined and implemented from the development and manufacture of pharmaceuticals, and has been described and recommended by the regulatory agencies [18]. Basically, its target is to design a product, considering its quality, that has the intended and consistent performance. QbD principles are increasingly being applied for the development of analytical methodologies, referred to as analytical QbD (AQbD), and its aim is to design a robust method that consistently delivers the desired performance [19]. This systematic approach used for the analytical development, based on the knowledge and risk assessment, increases the robustness, decreases the cost and provides a good regulatory flexibility [20,21,22]. The workflow applicable to AQbD is similar to the one used in QbD, which is described in ICH Q8 guideline [23]. The broad knowledge obtained from this process is used to define a method operable design region (MODR), a multidimensional space based on the method factors and settings that provide a suitable method performance [19,24]. In AQbD, the first stage is the definition of the method characteristics in combination with the intended purpose, called the analytical target profile (ATP); the analogous to quality target product profile (QTPP) is defined in QbD [19,25]. Normally, the ATP is expected to consider the regulatory requirements, such as ICH Q2 [26], assessing the quality characteristics needed for the methodology, such as the specificity, linearity, accuracy, precision, range, limit of detection (LOD) and limit of quantification (LOQ). Framing the ATP, all of the critical method attributes (CMeAs) are established, in combination with the selected acceptance criterion and specifications. Using a quality risk assessment approach, the critical method parameters (CMePs) are displayed as major influencing factors to the analytical method performance. It is important to establish experimentally the relationship between the CMeAs and CMePs, and also to assess the level of influence in the performance using a statistical design of experiments (DoE) [19,20,25,27,28,29].

**Figure 1:**
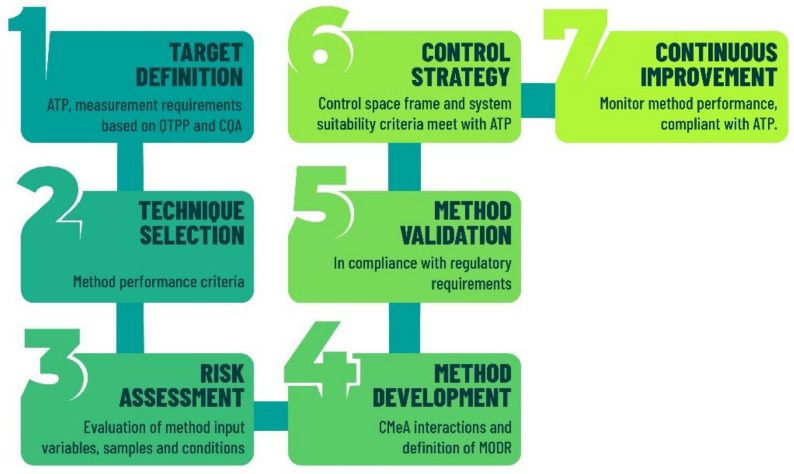
Implementation stages of AQbD, modified from Ramalingam and Jahnavi [30].

Eventually, the analytical methods developed using the AQbD approach could reduce the number of out-of-trend (OOT) results and out-of-specification (OOS) results due to the robustness of the method within the region [30]. Nowadays, it is trending within the pharmaceutical industry to implement AQbD in the method development process [30] as a part of risk management [31], pharmaceutical development [23] and the pharmaceutical quality system [22]. The stages for the implementation of AQbD into the method development are presented in Figure 1.

**Figure 2:**
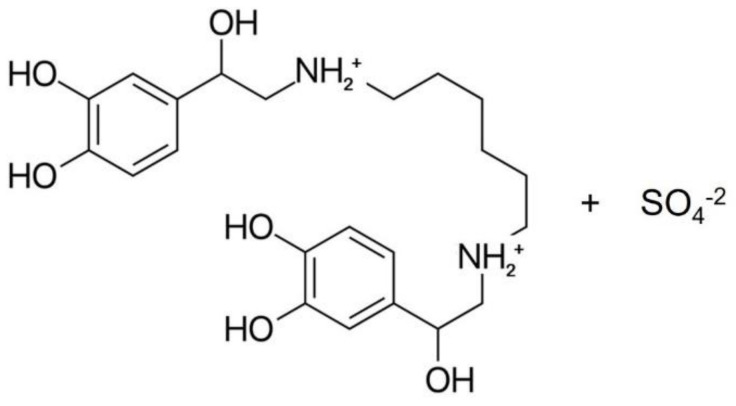
Molecular structure of hexoprenaline sulphate.

The hexoprenaline sulphate (Figure 2) has a pKa of 8.7 and a logP of 0.22 [32], which poses a challenge to the chromatographic retention, when using a typical reverse phase C18 column. Consequently, the use of ion-pairing reagents in the mobile phase would be ideal, in order to form an ion-pair between positively charged hexoprenaline, and a negatively charged ion-pair reagent through ionic interactions. This way, the overall polarity of hexoprenaline is reduced [33], which leads to an enhanced retention in the stationary phase.

## 2. Results and Discussion

### 2.1. Method Development by AQbD Principles

The ATP is a prospective summary of the requirements of a measurement system that, if achieved, will ensure an accurate assessment of a particular product quality attribute over the lifecycle of the product [19]. Using the chromatography methods as an analytical technique can fulfill the needed stability-indicating condition [13], and the UPLC was chosen as a suitable tool due to having a faster response and lower solvent consumption. The ATP shown in Table 1 assembles the internal and regulatory requirements for analytical methodologies [26,34]. The analytical method must be able to accurately quantify hexoprenaline in a range from 3.5 to 6.5 µg/mL with an accuracy of 100 ± 2% and a precision of ≤2% of the reportable value [35].

The hexoprenaline is a good candidate for elution by ion-pair (IP) chromatography. This technique has been traditionally performed with isocratic elution because increasing the organic modifier in the eluent results in a strong reduction of the IP reagent adsorbed on the stationary phase [36]. However, there are some examples of gradients used for the appropriate elution of polar compounds [37,38,39,40]. The gradient method is a good tool for achieving the separation of the related compounds that might appear during the degradation process of the product, having the stability-indicating feature that is required and intended [33,41].

For the method development experiments, the solutions of hexoprenaline sulphate were injected using several gradients. Variables such as columns, pH, gradient and ion-pair selection were adjusted to provide the best chromatographic and separation outcome i.e., resolution, tailing and efficiency. Afterwards, the developed analytical method was validated following the ICH Q2 (R1) guideline (Validation of Analytical Procedures) [26].

### 2.2. Definition of Critical Method Parameters (CMeP) and Critical Method Attributes (CMeA)

**Figure 3:**
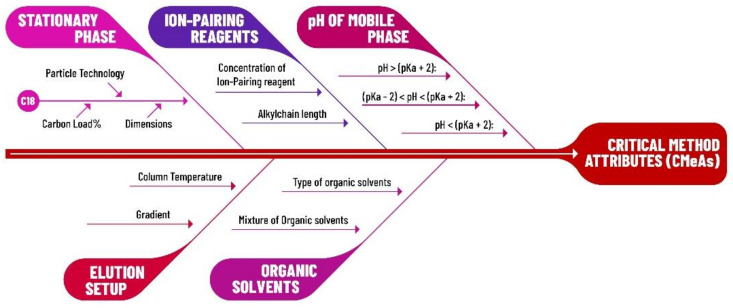
Proposed Ishikawa fishbone diagram for the assessment on CMeAs and CMePs, modified from Guiraldelli [42].

For the effective identification of the CMePs, a quality risk management (QRM) tool was used, such as the Ishikawa fishbone diagram, as shown in Figure 3. Using such a tool, it is possible to assess which method parameters are needed to be identified as critical due to their effect on the CMeAs.

Based on the materials available and the scope of this investigation, the screening design of experiment (DoE) for the CMePs are proposed in Table 2. The method is intended to be a fast elution UPLC run, so the length of the column was selected to be 50 mm. Both columns have the same C18 chemistry, but the T3 column differs from a C18 column in terms of the carbon coverage and ligand density. T3 columns have a lower carbon coverage, and they are less hydrophobic than a normal C18 column [43]. Acetonitrile was a preferred organic solvent, considering its lower relative toxicity than methanol [44]. Regarding the pH of the mobile phase, taking into account the ion-pairing dynamic, the levels of pH 3 and 4 were chosen, as recommended by literature and the supplier [36,45]. The development experiments were executed with fixed mobile phase “A” of sodium dihydrogen phosphate 5 mM + ion-pairing reagent sodium salt 10 mM, and acetonitrile as the organic modifier.

### 2.3. Statistical Evaluation of the Screening DoE

The following section provides the results of the statistical model, which was a least square regression fit. The statistical model was constructed on JMP^®^ Pro 14.2.0 software from SAS Institute Inc, and has the objective to find the relationship between the CMePs and the CMeAs. This interaction could be as an individual or combined interference on the response variable performance. The fit details, such as the “actual vs. predicted” plot and the “effect tests”, are provided separately for each of the y-responses: USP tailing, K prime, USP plate count, retention time and peak purity.

**Figure 4:**
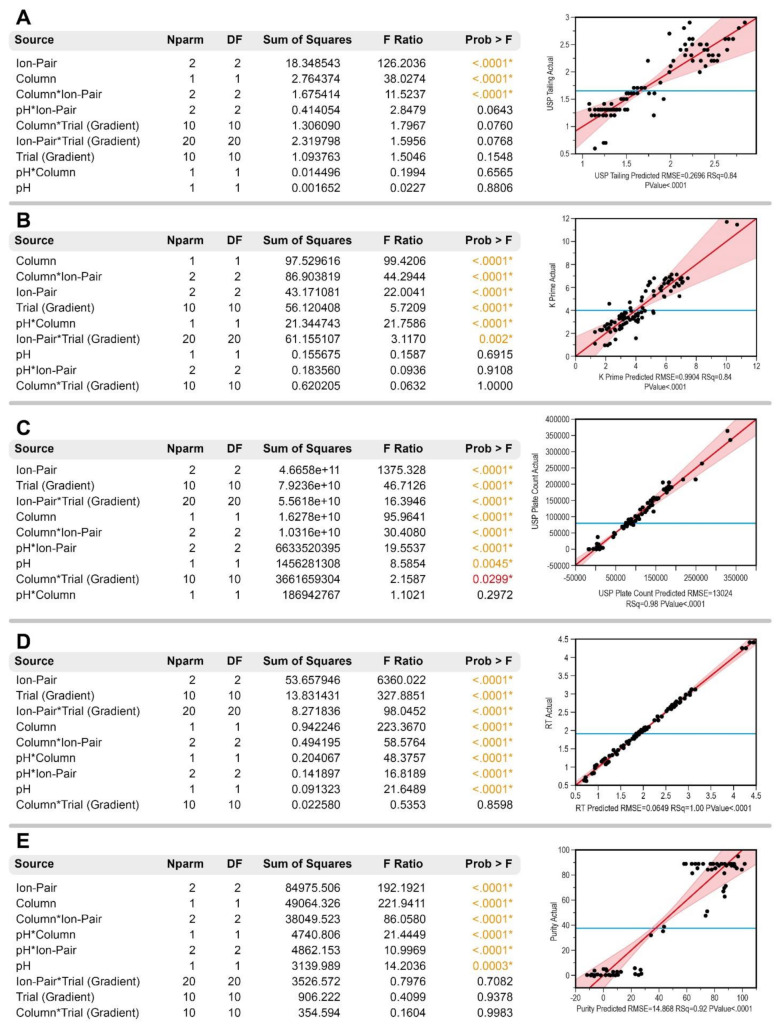
Effect tests (Being Nparm = number of parameters associated with this effect; DF = degree of freedom) and actual vs. predicted plot for USP tailing (**A**), K prime (**B**), USP plate count (**C**), retention time (**D**) and peak purity (**E**). Each black dot in the predicted vs. actual figure represents a data point with the mean (blue line) and 95% confidence interval (red shaded area around the red linear regression line).

The effect summary for the least square fit is shown in Table 3. In Figure 4, the actual vs. predicted plot for all of the chromatographic parameters indicates a good fit with R^2^ adjusted = 0.74 (A,B); R^2^ adjusted = 0.97 (C); R^2^ adjusted = 0.99 (D); R^2^ adjusted = 0.87 (E). The effect tests prove that the ion-pair reagent, and many interactions with the ion-pair reagent factor, have significant effects on the model (α = 0.05). The majority of experiments using methanesulphonic acid led to a lower retention time in the chromatographic column; thus not satisfying the proposed CMeAs. The retention time is an indicative measure connected with the USP plate count and K prime. This is expected, considering that, at a lower length of the alkyl chain in the ion-pairing reagent, there is a lower retention for the polar compounds on C18 stationary phases [36,42,46]. Regarding the peak purity (D), it was evaluated as the purity angle and purity threshold difference, where it is desirable to have a higher threshold, which indicates that there is no obvious co-elution within the range of the threshold angle, which indicates the effect of the noise [47].

Several interactions result in being significant for this model. The complex relationships between the pH, column, gradient and ion-pair agent were assessed and the ideal setup in terms of USP tailing, K prime, USP plate count, retention time and peak purity was defined. The desired value for each y-variable was defined as follow:

(1) USP tailing: Minimize (0.5 being the highest desirability value);
(2) K prime: Maximize (12 being the highest desirability value);
(3) USP plate count: Maximize (400,000 being the highest desirability value);
(4) Retention time: Match to target at 5 min;
(5) Peak purity: Maximize (100 being the highest desirability value).

**Figure 5:**
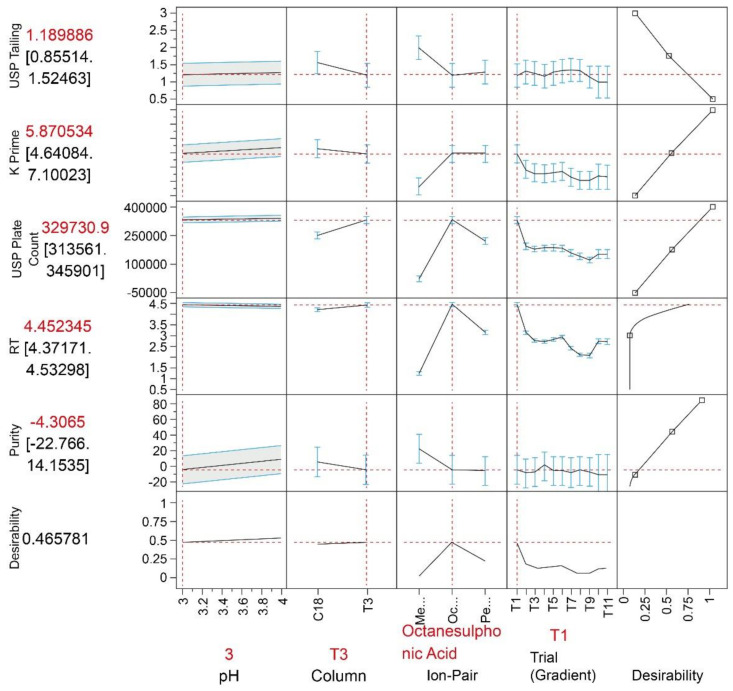
Prediction profiler with 95% confidence intervals. An overall desirability of 47% could be achieved with the setting illustrated.

With these settings being set a maximum, an overall desirability of 53% is achieved at the following setting: higher pH 4.0, T3 column, octanesulphonic acid and the first gradient experiment. However, considering the appropriate achieved K-factor for this chromatography (e.g., FDA requires K prime to be higher than 2 [34]), the USP tailing is considered to be constrained to be less than 2 [34]. Hence, by keeping the factors such as the column, ion-pair agent and gradient fixed while adjusting the pH to the lower studied level of 3.0, the desirability drops only by 6%. An accepted reduction of desirability is having the tailing factor shifted closer to 1. A graphical overview of the prediction profiler with a lower pH is shown below in Figure 5. The associated lower purity prediction values, however, indicated an issue with co-elution with this change. Experimental confirmation studies, however, confirmed pure peaks without co-elution using the setup described.

With the assessment of the chromatographic parameters, and the execution of the development experiments, it was sufficient to build a robust standard least square model. It is important to consider that DoE helps to identify and explain how CMePs affects the CMeAs and, therefore, the ATP [28]. Using the AQbD approach, a systematic and methodological experimentation led to the early identification of factors such as the ion-pair reagent, which is the utmost driver in the overall method performance. The predicted settings anticipate the experimental results, delivering a straight-forward approach for the method validation.

### 2.4. Final Method Conditions

According to the obtained results, the following conditions were selected as the final method conditions:

- Stationary phase: ACQUITY UPLC HSS T3 Column, 100 Å, 1.8 µm, 2.1 mm × 50 mm;
- Mobile phase A: sodium dihydrogen phosphate 5.0 mM + octane-1-sulphonic acid sodium salt 10 mM, adjusted with diluted phosphoric acid to a pH of 3.0;
- Mobile phase B: acetonitrile;
- Flow rate: 0.5 mL/min;
- Injection volume: 10 µL;
- Column temperature: ambient (25 °C);
- Detection wavelength: 280 nm;
- Gradient: t = 0 min, 5% B; t = 1 min, 5% B; t = 5 min, 50% B; t = 7 min, 5% B, t = 10 min, 5% B.

### 2.5. Validation of Analytical Method

#### 2.5.1. Specificity and Forced Degradation Studies

**Figure 6:**
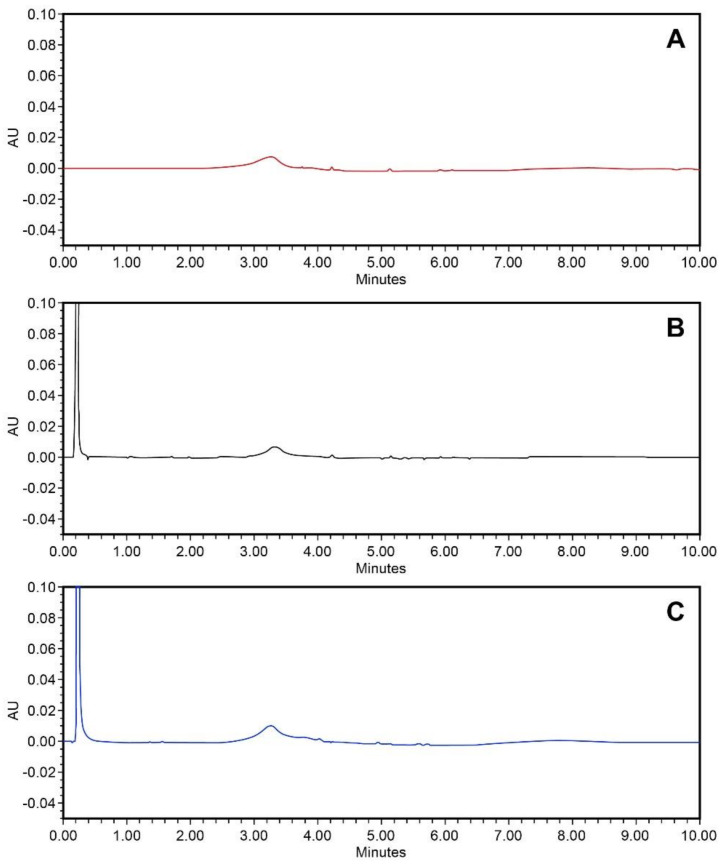
Chromatogram at 280 nm of mobile phase (**A**), blank (**B**) and placebo (**C**).

Specificity was tested on the mobile phase, diluent (blank), reference solution, matrix formulation components and final product. The analytical method has proven that it is selective for the quantification of hexoprenaline in the presence of the matrix. The chromatograms are presented in Figure 6.

The forced degradation study was conducted (see Table 4) and all of the peaks were eluted with sufficient resolution (more than 2) [34]. The chromatograms and purity plots of samples stressed under different conditions are depicted in Figure S1 (see Supplementary Materials). The hexoprenaline sulphate is prone to degradation by hydrolysis, and thus has been difficult to assess their degradation in a range that is still possible to have some un-degraded hexoprenaline. The injectable dosage form is stable under aqueous acidic conditions, which is also one of the reasons why the commercial product has a pH specification from 2.5 to 3.5 [48]. However, the hydrolysis increases under basic conditions, and thus, for the experiment, was adjusted in order to maintain sufficient un-degraded hexoprenaline. Under the photolysis and thermolysis study, there was no significant degradation. Under the oxidative stress condition, oxidation, as well as some hydrolysis, was expected, considering the water used as the reagent. The chromatograms were assessed in terms of peak purity and all of them were pure for all of the degradation conditions.

#### 2.5.2. Linearity, LOD/LOQ, Accuracy and Precision including Repeatability

**Figure 7:**
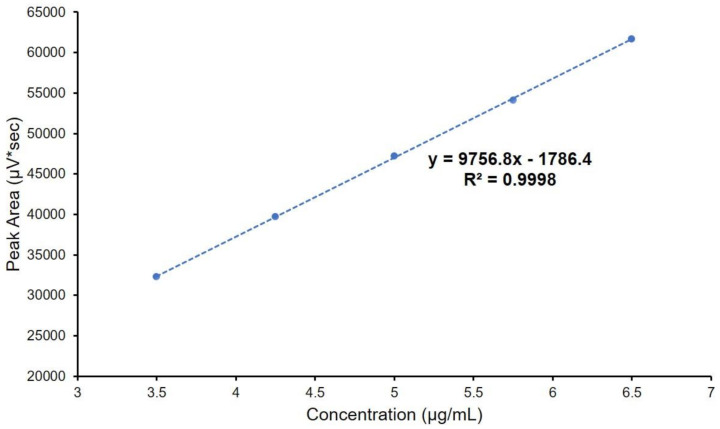
Graph representing hexoprenaline sulphate linearity results, including the linear equation.

**Figure 8:**
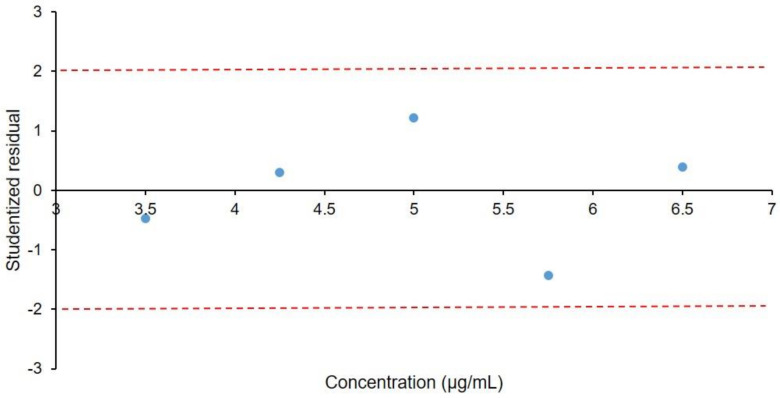
Studentized residual plot.

In Figure 7, the linearity is shown, and was proven from 3.50 µg/mL to 6.50 µg/mL for hexoprenaline sulphate (R^2^ = 0.9998). Additionally, Figure 8 presents the studentized residual plot, with no detected outliers or influence points; moreover, the points are within ±2 of the studentized residuals over the whole calibration range. The limit of detection (LOD) and limit of quantification (LOQ) were determined by the calculation of the S/N ratios of the prepared solutions. LOD and LOQ were determined to be 0.04 µg/mL and 0.12 µg/mL, respectively.

The accuracy was established based on the calculated recoveries at three concentration levels by triplicate. All of the recoveries were in the range of 100 ± 2% and are presented in Table 5.

**Figure 9:**
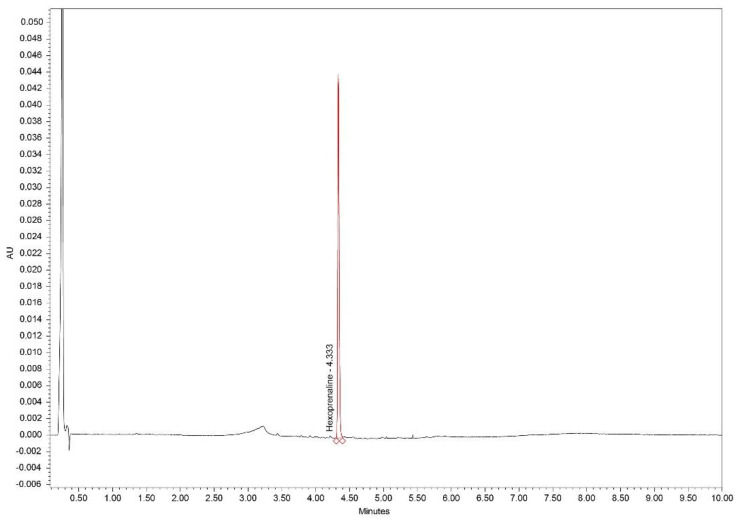
Chromatogram of hexoprenaline in Gynipral sample at 280 nm.

The developed method was found to be precise and reproducible for the analytes, since the RSDs for the repeatability and intermediate precision were below 2.0% (Table 6). An exemplary chromatogram of the sample is shown in Figure 9.

#### 2.5.3. Robustness

##### Stability of Solutions

The test, as well as standard solutions of hexoprenaline sulphate, was stable for at least 48 h (98–102%) at room temperature (mean recovery of 100.2% with a RSD of 0.7).

##### Column Temperature, Wavelength, Flow Rate and pH of Mobile Phase

The robustness of the analytical procedure in terms of the column temperature was determined by comparing the results of the samples in set conditions against variations of ±5 °C in the column temperature. The procedure is robust for hexoprenaline determination, in terms of the column temperature, since the individual values are within the range of ±2.0% and the mean recovery rate is within the range of 98.0 to 102.0%. There were no significant changes in chromatographic features defined as CMeAs. The procedure was robust, in terms of the wavelength detection, flow rate and pH of the mobile phase, since the individual values were within the range of ±2.0% and the mean recovery rate was within the range of 98.0 to 102.0%. The results are summarized in Table 7.

##### Column Variation

The robustness of the analytical procedure was assessed in terms of column variation by comparing the sample results of the defined column against a UPLC BEH C18 1.7 µm 2.1 × 50 mm, which was evaluated previously in the method development phase. Regarding the column variation, a mean recovery of hexoprenaline of 101.9% with a RSD of 0.2 was achieved. The procedure is robust for hexoprenaline determination, since the individual values are within the range of ±2.0% and the mean recovery rate is within the range of 98.0 to 102.0%. There were no significant changes in chromatographic features defined as CMeAs.

## 3. Materials and Methods

### 3.1. Reagents and Consumables

The acetonitrile used as solvent was purchased from VWR (Radnor, United States). The ion-pair reagents used were methanesulphonic acid anhydrous ≥ 98%, 1-pentanesulphonic acid anhydrous ≥ 98% from Loba Chemie (Mumbai, India) and 1-octanesulphonic acid monohydrate from Carl Roth (Karlsruhe, Germany). Regarding buffering agents used, sodium dihydrogen phosphate ≥ 99.0% and orthophosphoric acid 85% were purchased from VWR (Radnor, United States); sodium hydroxide ≥ 97% and hydrogen peroxide 30% were purchased from Sigma-Aldrich (St. Louis, MO, USA); hydrochloric acid 37% was purchased from Carl Roth (Karlsruhe, Germany); and sulfuric acid 96% was purchased from Merck (Darmstadt, Germany). The water used for all analyses came from the purification equipment 08.1205 from TKA Germany (Niederelbert, Germany). Buffered solution adjusted to pH 2.5–3.5 using sulfuric acid was used as diluent. All sample solutions were filtered before injecting into the chromatograph using nylon syringes filters (0.22 μm) from YETI Merz Brothers GmbH (Haid, Austria).

### 3.2. Standards, Samples and Excipients

The reference working standard of hexoprenaline sulphate (purity of 90.3%) was acquired from BOC Sciences (Shirley, NY, USA). The samples of hexoprenaline sulphate as injectable dosage form (10 µg/2 mL) (Gynipral^®^) were acquired from Takeda Austria GmbH (Linz, Austria). Sodium chloride, sodium disulfite from Merck (Darmstadt, Germany) and ethylenediaminetetraacetic acid disodium salt dehydrate from Sigma-Aldrich (St. Louis, MO, USA) were used as excipients in the used hexoprenaline product (Gynipral^®^), as per the patient information leaflet (PIL) [48].

### 3.3. Equipment

A Reversed Phase Ultra Performance Liquid Chromatograph H-Class from Waters Corp. (Milford, CT, USA) coupled with a photo-diode array detector (PDA) and equipped with the chromatographic software Empower 3 from Waters Corp. (Milford, CT, USA) was used for the method development, analysis and validation. The chromatographic columns used were from Waters Corp. (Milford, CT, USA): an Acquity UPLC BEH C18 (2.1 × 50 mm; 1.7 µm) and an Acquity UPLC HSS T3 (2.1 × 50 mm; 1.8 µm). The pH measurements were performed with pH-meter FiveEasy FE20 from Mettler Toledo (Columbus, OH, USA). The statistical analysis was performed on JMP^®^ Pro 14.2.0 software from SAS Institute Inc. (Cary, NC, USA).

### 3.4. Method Validation

#### 3.4.1. Specificity

The specificity of a method is its ability to unambiguously identify and separate the analyte in the presence of other components, such as degradation products, impurities, other active ingredients, excipients and matrix components [26]. Specificity was tested on mobile phase, diluent (blank), reference solution, matrix formulation components and final product. The chromatograms were recorded and evaluated.

#### 3.4.2. Forced Degradation Studies

In order to demonstrate the stability-indicating characteristics of the developed method, a forced degradation study was performed. Different amounts of hexoprenaline corresponding to 100% concentration were weighed and dissolved in the diluent (purified water with placebo) having a final concentration of 5 µg/mL of hexoprenaline sulphate (in triplicates). All solutions were prepared in 20 mL flasks and subjected to the following conditions:

- Photolysis: exposure to visible light for 8 h;
- Acid hydrolysis: exposure to 5.0 mL of hydrochloric acid (HCl) 1 N for 1 h;
- Basic hydrolysis: exposure to 1.0 mL of sodium hydroxide (NaOH) 0.5 N for 20 min;
- Oxidation: exposure to 5.0 mL of hydrogen peroxide (H_2_O_2_) 30% for 1 h;
- Thermolysis: exposure to heat (70 °C) in a steam bath for 1 h.

In the cases of acid and basic hydrolyses, once the exposure time was over, samples were neutralized with NaOH and HCl solutions, respectively, and filled with diluent solution. The possible degradation was screened by comparing the obtained chromatograms with that of the control sample. These analyses were completed with the purity study of the chromatographic peak.

#### 3.4.3. Linearity

The linearity of an analytical procedure shows that the obtained results are directly proportional to the relevant concentration range of the analyte [26]. Five solutions at concentrations between 70 and 130% of the declared content/labelled claim were prepared by dilution, dissolving in the purified water spiked with placebo [35].

#### 3.4.4. Accuracy

The accuracy of an analytical procedure gives an indication of systematic uncertainties in results. It is the degree of agreement between the expected value or the reference value and the value obtained [26]. Solutions of hexoprenaline sulphate at three concentrations levels of 70%, 100% and 130% of the declared content/labelled claim were prepared by weighing and dissolving in the diluent and further analyzed. The samples also contained the same excipients as declared in the commercial formulation [48].

#### 3.4.5. Precision (Repeatability and Intermediate Precision)

The precision of an analytical procedure expresses the closeness of agreement (degree of scatter) between a series of measurements obtained from multiple sampling of the same homogeneous sample under the prescribed conditions. Repeatability expresses the precision under the same operating conditions over a short interval of time. Repeatability is also termed as intra-assay precision [26]. The repeatability was investigated by analyzing six independent determinations of the final dosage form (*n* = 6). The intermediate precision was determined using a second test series of identically prepared samples. The reagents and samples were freshly prepared and analyzed by a second analyst. The degree of difference was assessed.

#### 3.4.6. Robustness

The robustness of an analytical process defines its resilience to small but intended changes in the method parameters and thus provides information on the reliability of the method in routine operation [26,35]. The effects of the stability of solutions were assessed for 48 h at room temperature condition (20 °C), and changes in the chromatographic column temperature (±5 °C), injection volume (±2 µL), flow rate (±0.1 mL/min), mobile phase pH (±0.2), different chromatographic column (ACQUITY UPLC BEH C18 and ACQUITY UPLC HSS T3) and wavelength of quantification (±2 nm) on the obtained results were evaluated. The results were compared with those of repeatability test. Additionally, the chromatographic parameters from the system suitability were assessed.

## 4. Conclusions

A new stability-indicating reversed-phase UPLC method for the determination of hexoprenaline was developed using AQbD principles. A statistical evaluation was conducted, resulting in a least square regression fit model for the assessment of CMeAs with regard to the CMePs. It was possible to build a better discernment on the effect of the method parameters on the target performance of the method. The proposed analytical methodology was validated according to ICH Q2 guidelines, confirming to be linear, accurate, precise, specific and robust. This method is a good example of how the QbD tools are applicable for the development of analytical methodologies, in a pragmatic manner, providing a better understanding of the procedures, increasing the data quality and decreasing the costs. AQbD completes the overall lifecycle concept of analytical methods and is highly robust, easily validated and cost- and time-effective because less experimental work is required for the method development [49].

## Figures and Tables

**Table 1:** Analytical target profile for hexoprenaline assay of injectable dosage form.

Analytical Methodology	Expected Method Response (CMeAs)
UPLC with PDA detection	Tailing factor ≤ 2 Resolution > 2 Capacity factor (k′) > 2 Plate count > 20,000 RT = 3–6 min Acceptable peak purity

**Table 2:** Initial screening DoE for the assessment of CMePs.

Factors →	Ion Pairing Reagent	Column	pH of Mobile Phase	Gradient
Levels →	Methasulphonic acid Pentasulphonic acid Octanosulphonic acid	ACQUITY UPLC BEH C18 column, 130 Å, 1.7 µm, 2.1 mm × 50 mm ACQUITY UPLC HSS T3 column, 100 Å, 1.8 µm, 2.1 mm × 50 mm	3.0 4.0	9 gradient combinations

**Table 3:** Effect summary for the whole model using a factorial standard least square method up the 2nd degree with only significant terms.

Source	LogWorth		*p* Value
Ion-Pair	82.798	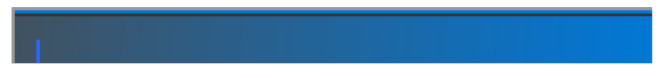	0.00000
Experiment (Gradient)	56.311	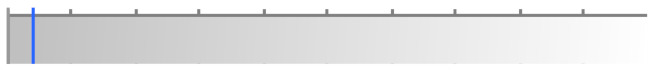	0.00000
Ion-Pair*Exp. (Gradient)	44.353	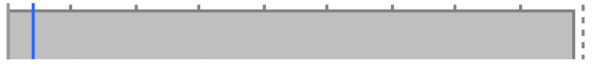	0.00000
Column	23.324	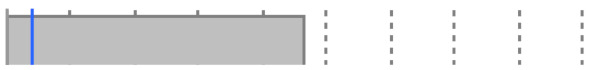	0.00000
Column*Ion-Pair	19.311	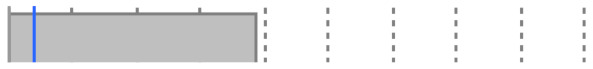	0.00000
pH*Column	8.924	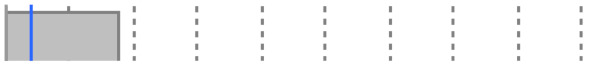	0.00000
pH*Ion-Pair	6.818	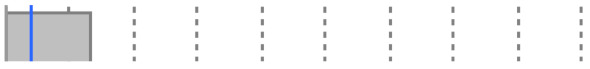	0.00000
pH	4.853	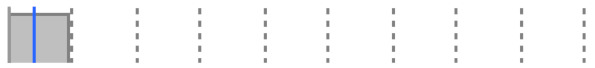	0.00001
Column*Exp. (Gradient)	1.524	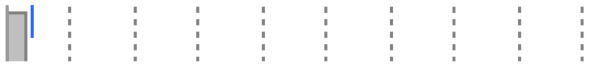	0.02991

**Table 4:** Results of forced degradation studies on hexoprenaline.

Condition	% Hexoprenaline Degradation (mean + std.dev.)
Photolysis	0.7 ± 0.3
Acid hydrolysis	3.9 ± 1.4
Basic hydrolysis	36.9 ± 1.3
Oxidation	9.5 ± 1.1
Thermolysis	0.6 ± 0.0

**Table 5:** Accuracy results from method validation.

Percentage of Target (%)	Hexoprenaline	
	Average (%)	RSD (%)
**70%**	99.8	0.2
**100%**	99.7	0.1
**130%**	101.0	0.1

**Table 6:** Precision results from method validation.

Analyst	Hexoprenaline	
	Average (%)	RSD (%)
I	99.7	0.1
II	101.9	0.2
Absolute difference (%)	2.2	

**Table 7:** Robustness results from method validation.

Condition	Hexoprenaline	
	Average (%)	RSD (%)
Col. Temp. (20 °C)	100.4	0.2
Col. Temp. (30 °C)	100.4	0.2
Wavelenght 278 nm	100.2	0.1
Wavelenght 282 nm	100.2	0.1
Flow rate 0.4 mL/min	100.9	1.2
Flow rate 0.6 mL/min	100.3	0.2

## Data Availability

The data presented in this study is available within the research and supplementary material of the article.

## Supplementary Materials

The following are available online, Figure S1: Chromatograms and Purity plots of hexoprenaline forced degradations on acid (A), alcaline (B), oxidative (C) and thermal (D) conditions, Figure S2: Regression standardized residuals, Table S1: Evaluation of robustness on system suitability test.
