# Supplementary material for: Development and Validation of a Stability-Indicating UPLC Method for the Determination of Hexoprenaline in Injectable Dosage Form Using AQbD Principles
Source: Molecules. 2021 Oct 31;26(21):6597. doi: 10.3390/molecules26216597 (PMC8587854; doi:10.3390/molecules26216597)
Supplement: Supplementary file 1 [file molecules-26-06597-s001.zip › molecules-1382390-supplementary.pdf]

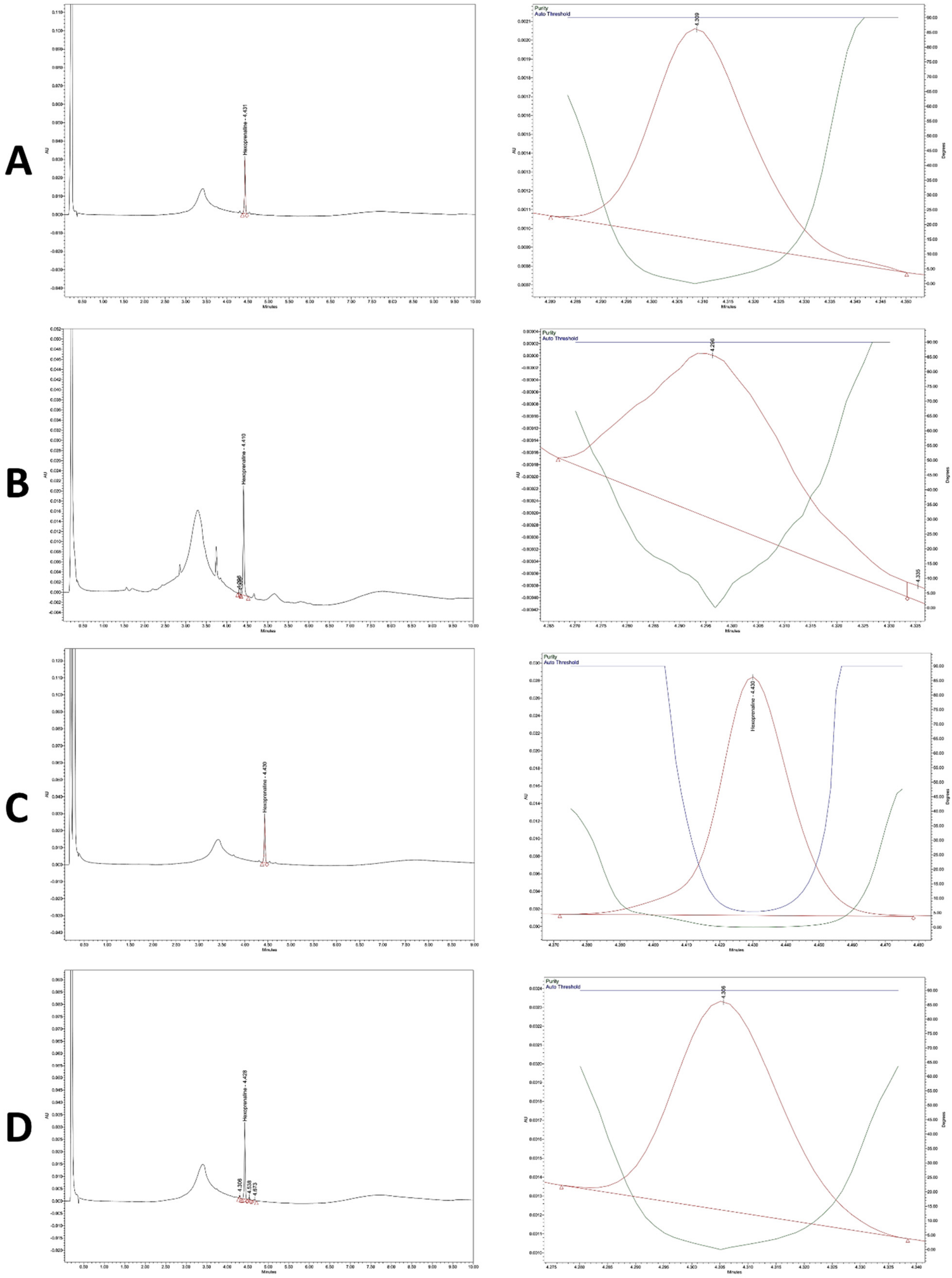

Figure S1. Chromatograms and Purity plots of samples exposed to forced degradations acid (A), alcaline (B), oxidative (C) and thermal (D).

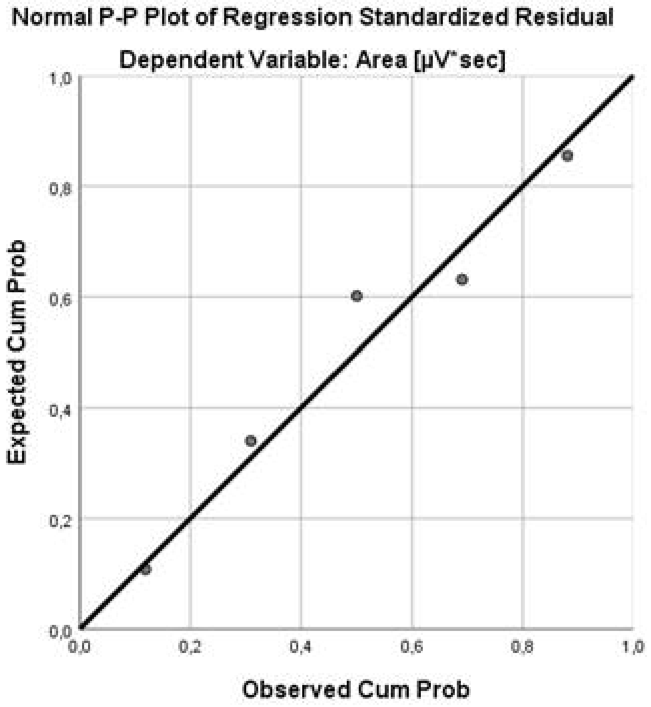

Figure S2. Regression standardized residuals.

Table S1. Evaluation of robustness on system suitability test

Chromatographic feature	Original conditions	Colum varianti on	Column temperature		Flow rate		pH of mobile phase		Wavelenght	
			20°C	30°C	0.4 ml/min	0.6 ml/min	2.8	3.2	278 nm	282 nm
%RSD (5 inj.)	0.2	0.0	0.2	0.1	1.3	0.1	0.2	0.2	0.1	0.1
Plate count	172431	196001	168016	181817	172150	166082	256497	257123	176735	176675
Tailing factor	1.42	1.30	1.40	1.40	1.40	1.40	1.20	1.20	1.40	1.40
RT	4.38	4.33	4.39	4.37	4.71	4.14	4.36	4.38	4.38	4.38
